# REcovery from DEXmedetomidine-Induced Unresponsiveness (REDEX): A Study Protocol for a Single Center, Parallel Arm, Non-Randomized, Controlled Pilot Trial in Healthy Volunteers

**DOI:** 10.2147/NSS.S523111

**Published:** 2025-06-12

**Authors:** David R Schreier, Matteo Fecchio, Christian S Guay, Reid G Kovacs, Mark Olchanyi, Ariel L Mueller, Timothy T Houle, Brian L Edlow, Emery N Brown, Ken Solt

**Affiliations:** 1 Department of Anesthesia, Critical Care and Pain Medicine, Massachusetts General Hospital and Department of Anaesthesia, Harvard Medical School, Boston, MA, USA; 2 Picower Institute for Learning and Memory, Massachusetts Institute of Technology, Cambridge, MA, USA; 3 Center for Neurotechnology and Neurorecovery, Department of Neurology, Massachusetts General Hospital and Harvard Medical School, Boston, MA, USA; 4 Department of Neurology, Inselspital, Bern University Hospital, and University of Bern, Bern, Switzerland; 5 Department of Anesthesiology, Perioperative and Pain Medicine, The University of Utah, Salt Lake City, UT, USA; 6 Institute for Medical Engineering and Science, Massachusetts Institute of Technology, Cambridge, MA, USA; 7 Department of Brain and Cognitive Sciences, Massachusetts Institute of Technology, Cambridge, MA, USA; 8 Department of Electrical Engineering and Computer Science, Massachusetts Institute of Technology, Cambridge, MA, USA; 9 Athinoula A. Martinos Center for Biomedical Imaging, Massachusetts General Hospital, Charlestown, MA, USA

**Keywords:** consciousness, sedation, sleep, electroencephalography, transcranial magnetic stimulation, target-controlled infusion

## Abstract

**Purpose:**

Dexmedetomidine (DEX) is a well-tolerated sedative drug that induces a sleep-like state. DEX sedation offers a model to study transitions between different states of consciousness (indicated by, eg, behavior, the electroencephalogram (EEG), or transcranial magnetic stimulation (TMS) evoked EEG responses). However, the effects of repeated DEX exposure on recovery are poorly understood and will be investigated in this pilot study.

**Participants and Methods:**

We aim to enroll 12 healthy volunteers (6 females, 6 males). Although we do not expect TMS-EEG to interfere with DEX sedation, due to the paucity of evidence this study uses a parallel arm design (TMS-EEG, non-TMS-EEG). Participants will be sedated twice, one week apart, and responsiveness monitored by a click-task to auditory beeps. A 64-channel EEG and additional physiological signals will be recorded. Cognition and vigilance tests will be performed before sedation (baseline), after return of responsiveness (ROR), and before discharge. TMS-EEG will be performed at baseline, during sedation, and during recovery. Using a smartwatch and questionnaires, we will assess sleep quality, sleepiness, and experiences during sedation and TMS-EEG.

**Results:**

We will report the difference of time to ROR between the first and second study visit, and explore potential differences across sex and study arms. We will evaluate state transitions by comparing responsiveness, traditional EEG signatures, TMS-evoked EEG responses (ie, perturbational complexity index), and report on cognition and vigilance test performance.

**Conclusion:**

This pilot trial will report on the effect of repeated DEX exposure on the recovery period, and the investigation of state transitions will advance our scientific understanding of altered states of consciousness. REDEX will provide valuable insights and data for designing future DEX sedation studies. Moreover, we will report on the potential of biological sex as a confounding factor and the feasibility of TMS-EEG under DEX.

## Introduction

### Dexmedetomidine and Sleep

Dexmedetomidine (DEX) is a highly selective α_2_-adrenergic-receptor agonist, known for its sedating effect creating a sleep-like state (behavioral unresponsiveness but rousable, with preserved respiration).^1–3^ Although the recovery from DEX sedation is slow (in-human half-life ≈ 2h) and DEX has hemodynamic side effects, such as hypotension and bradycardia, the high α2-receptor affinity of DEX contributes to a more favorable overall hemodynamic and sedative profile compared to other α2-adrenergic-receptor agonists, such as clonidine.^1^,^4–6^ Additional advantages of DEX include analgesic effects that reduce postoperative opioid requirements, lower rates of postoperative delirium compared to other anesthetics, and electroencephalogram (EEG) features that are similar to those of natural sleep.^5^,^7–10^ Therefore, DEX is widely used in operating rooms (OR) and intensive care units (ICU).

DEX induces a sleep-like state through inhibition of noradrenergic neurons in the locus coeruleus that project to the preoptic area, thalamus, and cerebral cortex.^11^,^12^ The mechanistic similarity to non-REM sleep (NREM), also involving the locus coeruleus, is reflected behaviorally (arousability and preserved respiration) and in the EEG. At lower doses, DEX prominently induces NREM Stage 2 (NREM-2) like spindles; at higher DEX doses, these fade away while power in the delta band increases, similar to NREM Stage 3 (NREM-3).^13^,^14^ To which extent DEX is inducing states that mimic natural sleep (biomimetic sleep), whether it induces – at least partially – natural sleep, or if DEX sedation itself induces restorative processes in the brain is unknown.

### Repeated Exposure to Dexmedetomidine

The similarities between DEX and natural sleep, including preserved respiration and rousability, make DEX sedation an intriguing model for studying altered states of consciousness, the corresponding transitions, and interventional studies under highly controlled experimental conditions. However, the complexity, labor and infrastructure requirements, as well as the high costs are limiting the feasibility of interventional trials using such a sedation model, especially when including human volunteers. An optimized study design, eg, choosing a cross-over instead of a parallel arm study design, can reduce the sample size without losing statistical power but only if the exposure to DEX, followed by an adequate washout period, does not affect outcomes of future experiments with exposure to DEX.

While many aspects of DEX sedation are unknown, and only a few of which can be addressed within the scope of a pilot trial, the main focus of this study is to investigate if repeated exposure to DEX has an effect on the recovery period and more specifically the time to return of responsiveness (ROR).

### Biological Sex and Time to Return of Responsiveness

Although sex differences can have implications for patient care and scientific studies, there has been a paucity of research investigating the effect of sex on sensitivity to anesthetic drugs in humans.^15^ A recent animal study found that the time to emergence from DEX was significantly longer in female rats compared to males.^16^ The timepoint of emergence in rats is commonly defined by the return of the righting reflex and used as a correlate of return of responsiveness in humans. A follow-up study using female rats showed that differences in time to emergence were related to the rodent estrous cycle,^17^ and some human evidence suggests also an influence of the menstrual cycle on recovery from DEX in humans.^18^ If the menstrual cycle has an effect on time to ROR, this might introduce bias when aiming to determine the effect of repeated exposure. Assuming that a potential effect of the menstrual cycle stems from hormonal fluctuations, the interaction with DEX and time to ROR should be minimal or non-existent in females that either take oral hormonal contraception or are postmenopausal. Consequently, and assuming that there is no relevant hormonal impact on time to ROR in males, we will limit the inclusion criteria of females in this study to those who use oral hormonal contraception or are postmenopausal in order to minimize the impact of biological sex as a confounding factor.

### Capacity for Consciousness and Higher-Order Brain Functions

Although the primary outcome variable of this study is based on responsiveness (time to ROR), understanding the formation and dissolution of consciousness when unresponsive is one of the great challenges in neuroscience and key motivators to use a sedation model.^19^ In addition to classic EEG signatures suggesting unconsciousness, EEG based measures of complexity, such as the perturbational complexity index (PCI), are increasingly popular to determine the capacity for consciousness when behaviorally unresponsive.^20–25^ The PCI conceptually determines the amount of information contained in the integrated response of the thalamocortical system to a direct perturbation (eg, TMS).^20^ The TMS-EEG associated risks are minimal, and both DEX sedation and the prolonged recovery from DEX are a scientific opportunity to study and characterize different states of consciousness.^26^ To date, only one study in humans has performed TMS-EEG during DEX sedation.^25^ Cardone et al demonstrated that stimulating frontal cortical areas during sedation results in higher amplitudes of TMS-evoked potentials than at baseline. In contrast, the amplitude of TMS-evoked potentials to stimulation of parietal cortical areas did not change between conditions. The effect of (repeated) DEX sedation on TMS-EEG derived measures of complexity across different cortical areas is unknown, especially during unresponsiveness. Cardone et al reported that 35% of participants were still responsive to verbal commands at the maximum predicted effect-site concentration (2.5 ng/mL). Although we believe that these participants would have required higher doses of DEX to become unresponsive and that this was not an arousal-promoting effect of TMS-EEG, this study will have a TMS-EEG arm and a non-TMS-EEG arm. Moreover, the maximum predicted DEX effect-site concentration allowed in this study will be higher (4 ng/mL), and the targeted effect-site concentration will be based on loss of responsiveness (LOR + 0.5 ng/mL). We will report measures of complexity for different cortical sites and conditions (at baseline, during sedation, and during the recovery period). In addition to using TMS-EEG to assess the capacity for consciousness and its change over the course of the experiment, this study will also use cognition and vigilance tests to monitor the recovery of higher-order brain functions from DEX sedation.

### Specific Aims

The primary aim of this study is to determine the effect of repeated exposure on time to ROR from DEX-induced unresponsiveness. We hypothesize that time to ROR will not significantly differ between the first and second exposure in the same individuals, and that inter-individual differences will be larger than intra-individual differences, respectively. Although we aim to minimize the potentially confounding effect of hormonal fluctuations in females by adjusting our inclusion criteria accordingly and we assume that TMS-EEG performed at cortical sites does not have an arousal-promoting effect, we will explore potential effects of biological sex or TMS-EEG on time to ROR.

The secondary aims of this study are: (i) to investigate the relationship between measures of responsiveness, EEG signatures suggesting unconsciousness, and measures of complexity in the TMS-EEG arm; (ii) to characterize the recovery from DEX exposure for higher-order brain and autonomic functions.

## Materials and Methods

The reporting of this study protocol follows the ‘Standard Protocol Items: Recommendations for Interventional Trials’ (SPIRIT) 2013 statement.^27^ The study was approved by the Institutional Review Board (IRB) of the Massachusetts General Hospital (MGH), Boston, MA, USA, will follow the principles of the Declaration of Helsinki,^28^ and is registered on ClinicalTrials.gov (NCT06003127). This single center, parallel arm, non-randomized, non-blinded, controlled pilot trial in healthy volunteers will be performed at MGH in line with institutional and legal regulations and Good Clinical Practice (GCP). Amendments of the originally approved protocol will be submitted to the MGH IRB, and the ClinicalTrials.gov entry will be updated whenever appropriate.

### Participants, Interventions, and Outcomes

#### Timeline, Recruitment, and Sample Size

The planned period for the data collection is 12–24 months, followed by an additional 12 months for data analysis. We will recruit healthy volunteers by advertisement through official study recruitment channels of Mass General Brigham (MGB) and through cross-recruiting from another MGH study (MGH IRB #2019P003811). Similar to other pilot studies, this study is constrained by limited prior information and feasibility considerations. Therefore, and as outlined by Steven A. Julious, the optimized sample size for this study is 12.^29^ To ensure balanced representation of biological sex, we will assign three female and three male healthy volunteers to each study arm.

#### Eligibility Criteria

We will enroll healthy volunteers in accordance with the inclusion and exclusion criteria outlined in Table 1. Volunteers that pass a pre-screening phone call will be scheduled for a screening visit. The goal of the screening visit is to determine study eligibility, obtain informed and written consent, and ensure that participants are enrolled in an appropriate health care plan to ensure coverage in the case of an adverse event requiring follow-up medical care.

**Table 1:** Eligibility Criteria

Inclusion criteria
18 to 65 years old BMI 18–30 kg/m^2^ ASA status = 1 For females: either use of hormonal contraception, or >45 years old and last menstrual period >12 months ago in the absence of any contraceptives. Fluent in English (sufficient to communicate with the study team, to understand the consent form, and to understand cognition and vigilance test instructions)
**Exclusion criteria**
Smoking History of taking stimulants or substance abuse ASA status > 1 Positive urine drug screening test Chronic health conditions (not limited to): **Neurologic**: Epilepsy or positive history of a seizure, stroke, central disorders of hypersomnolence, neuroimmunological disorder (eg, multiple sclerosis), Meniere’s disease, Parkinson’s disease, peripheral neuropathy, no significant visual or hearing impairments, findings in the clinical examination suggesting a neurologic disorder. **Psychiatric**: History or treatment for an active psychiatric problem (including ADHD and anxiety disorder). **Cardiovascular**: Hypertension, symptomatic hypotension or bradycardia, myocardial infarction, coronary artery disease, peripheral vascular disease, dysrhythmias, congestive heart failure, cardiomyopathy, valvular disease, familial history of sudden cardiac death **Respiratory**: Bronchitis, asthma, chronic obstructive pulmonary disease, smoking, shortness of breath, sleep apnea. **Gastrointestinal**: Esophageal reflux, hiatal hernia, ulcer **Hepatic**: Hepatitis, jaundice, ascites **Renal**: Acute or chronic severe renal insufficiency **Reproductive**: Pregnancy, breast-feeding **Endocrine**: Diabetes, thyroid disease, adrenal gland disease **Hematologic**: Blood dyscrasias, anemia, coagulopathies **Musculoskeletal**: Prior surgery or trauma to head neck or face, arthritis, personal or family history of malignant hyperthermia. **Medications**: Regular use of prescription and non-prescription medications expected to affect central nervous function, anticoagulant or thrombocyte-aggregation inhibiting therapy; exception: oral hormonal contraception. **Allergies**: Dexmedetomidine, phenylephrine, betablockers, hydralazine, glycopyrrolate.

**Abbreviations**: ADHD, attention-deficit/hyperactivity disorder; ASA status, American Society of Anesthesiologists physical status class risk;^30^ BMI, Body Mass Index.

Following informed consent, we will take a complete medical history and determine TMS-EEG eligibility. Females will additionally receive a short questionnaire (asking about the menstrual cycle and the use of contraception) to determine eligibility and for scheduling study visits outside of the menstrual period. A complete physical examination will be performed, and abnormal findings will be reported to the potential participant and may result in exclusion from the study. Laboratory values derived from a standard blood draw must be within ±10% of the reference range, and a 12-lead electrocardiogram (ECG) must not show any contraindications for sedation. A urine sample will be tested for recreational drug use in all participants, and we will test for pregnancy in females (hCG). The urine drug screening, pregnancy testing and eligibility for TMS-EEG, if applicable, will be repeated at the beginning of each study visit and results will be reported to the participant.

#### Study Arm Allocation

**Figure 1:**
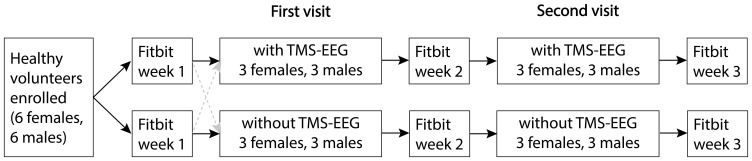
Parallel arm study design. Non-randomized assignment of healthy volunteers with the option for reassignment (dashed arrows) prior to data collection during the first study visit. The first and second study visit are about one week apart. Fitbit data collection over three weeks.

**Abbreviations**: TMS-EEG, transcranial magnetic stimulation electroencephalography.

Because of the higher logistical demand and complexity of scheduling TMS-EEG, we will assign study participants primarily to the TMS-EEG study arm at first. Such an approach enables us to reassign participants to the non-TMS-EEG study arm in case of unavailability of the TMS-EEG staff, a participant’s preference to not undergo TMS-EEG, or in case of new contraindications (eg, incompatible piercings/implants in the head region; Figure 1). We will ensure allocation of an equal number of females and males to each study arm eventually.

#### Interventions

To ensure that participants feel well rested for the experiment and minimize the potentially confounding effect of sleep deprivation, we will ask participants to follow a regular sleep-wake rhythm with sufficient sleep starting one week prior to the first study visit up until one week after the second study visit (Figure 1). They will receive a sleep-wake diary and a smartwatch to monitor their sleep-wake rhythm. We will also instruct participants to restrict alcohol consumption to one drink per day or less (National Institutes of Health dietary guidelines for Americans) in the week prior to each study visit. Moreover, participants will be required to avoid alcohol on the day before the visit and have nothing to eat on the day of the study visit. They will be allowed to drink water and clear liquids up to 2 hours before arrival, which will be in the morning.

**Figure 2:**
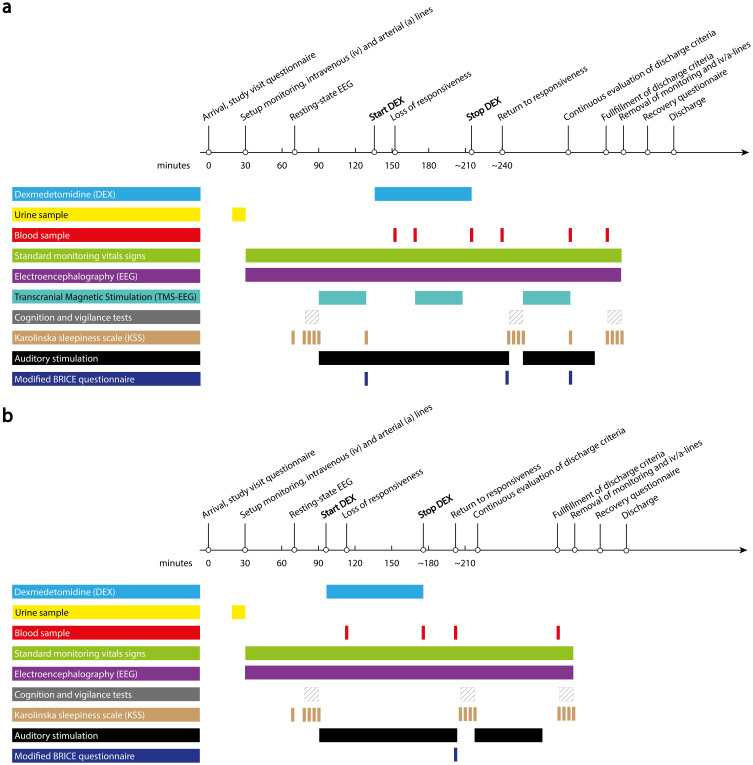
Overview of study visit procedures for (**a**) the transcranial magnetic stimulation electroencephalogram (TMS-EEG) study arm and (**b**) the non-TMS-EEG study arm. A modified version of the BRICE questionnaire will be used to ask about any experiences.^37^

**Abbreviations**: DEX, dexmedetomidine; EEG, electroencephalography; iv/a-lines, intravenous/arterial lines; TMS-EEG, transcranial magnetic stimulation electroencephalography; KSS, Karolinska Sleepiness Scale.

For sedation with DEX, we will use a target-controlled infusion (TCI) consisting of a Harvard 22 syringe pump and Rugloop II©, a Windows^®^ based TCI software that uses validated pharmacokinetic models to achieve stable plasma concentrations.^31–35^ The first targeted effect-site concentration will be 4 ng/mL, and the infusion rate will be limited to 6 mcg/kg/h. Oxygen supplementation will be delivered by face mask or nasal cannula. To monitor responsiveness, study participants will be instructed to click a button in response to auditory stimulation (a standardized beep) which will be delivered every 5 seconds starting prior to induction and stopped after ROR (in the TMS-EEG study arm after the post-sedation TMS-EEG session). We define LOR as failure to click the button five consecutive times in response to auditory beeps. If LOR does not occur within 30 minutes of DEX sedation, the maximum infusion rate of 6 mcg/kg/h will be reduced to minimize the risk of symptomatic bradycardia.^33^,^35^,^36^ Based on previous evidence, we anticipate LOR to occur mostly at effect site concentrations between 1.5 and 2 ng/mL.^31^ To achieve a stable state of sedation, we will target an effect-site concentration that is 0.5 ng/mL higher than the effect-site concentration at LOR, with a maximum of 4 ng/mL. We will maintain the targeted effect-site concentration for 60 minutes and then stop the DEX-infusion (Figure 2). To retrospectively compare TCI-estimated DEX concentrations with DEX plasma levels, we will take blood samples at multiple time points (Figure 2). We define ROR as five consecutive click responses to auditory beeps after stopping DEX. Click responses will be automatically marked in the EEG recordings and muscle activity of the forearm co-registered (electromyography, EMG).

We will additionally record ECG, pulse oximetry, capnography, plethysmography, galvanic skin response, and invasive blood pressure measurement (with an arterial catheter) to avoid a potential arousing effect of non-invasive blood pressure measurement. We will record baseline periods both in an upright and lying position. Frequent and expected side effects will be managed by a dedicated anesthesiologist in accordance with pre-defined and standardized scenarios (Table 2). If the pre-defined actions do not resolve the issue or if any other safety concerns arise (eg, signs of allergic reaction), DEX sedation will be terminated immediately. Additional medication may be used at the discretion of the anesthesiologist, and additional medical care administered (with the full support of the study team).

**Table 2:** Standardized Scenarios for Frequent and Expected Side Effects

HR Scenario	Action		If DEX > 1.5 mcg/kg/h
For 1 min: HR < 40/min For 2 min: HR < 50/min	Glycopyrrolate 0.2–0.4 mg		Discontinuation of DEX
For 1 min: HR > 140/min For 2 min: HR > 120/min	Esmolol 10–30 mg		Discontinuation of DEX
**SpO2 scenario**	**Airway free**	**Airway obstruction**	
For 1 min: SpO2 < 90% For 2 min: SpO2 < 95%	Increase oxygen flow to 10 liters/min	Airway maneuvers (chin lift, jaw thrust) followed by oral airway. If these maneuvers do not rescue desaturation: stop DEX, oxygenation with Ambu bag and 100% FiO2.	Discontinuation of DEX
**BP scenario**	**HR < 60/min**	**HR > 60/min**	
For 1 min: BP systolic < 90 mmHg BP diastolic < 50 mmHg For 2 min: BP systolic < 100 mmHg BP diastolic < 60 mmHg	Phenylephrine 80–160 mcg Glycopyrrolate 0.2–0.4 mg	Phenylephrine 80–160 mcg	Discontinuation of DEX
For 1 min: BP systolic > 180 mmHg BP diastolic > 120 mmHg For 2 min: BP systolic > 160 mmHg BP diastolic > 100 mmHg	Hydralazine 5–20 mg	Labetalol 5–50 mg	Discontinuation of DEX

**Abbreviations**: BP, blood pressure; DEX, dexmedetomidine; FiO2, fraction of inspired oxygen; HR, heart rate; SpO2, oxygen saturation.

TMS-EEG will be performed before, during, and after sedation, ie, after ROR (Figure 2). We will primarily focus on premotor and parietal cortical sites. To ensure the presence of a TMS-evoked response and to optimize the signal-to-noise ratio, the stimulation intensity will be adjusted on an individual basis before sedation. The target range of the evoked electric field intensity will be 100–150 V/m and the upper limit at 160 V/m. Throughout the TMS-EEG session, we will continuously monitor the EEG and masking noise will be delivered through over-ear headphones to prevent participants from hearing the clicking sound produced by the TMS.^38^ This will not interfere with the in-ear headphones delivering auditory beeps for monitoring responsiveness and we will ensure that noise levels will not exceed a maximum of 85 dB SPL, as recommended by the National Institute for Occupational Safety and Health.^39^ We will document any arousal or other events of relevance during the TMS-EEG session and, at baseline and during recovery, ask participants about any experience throughout the TMS-EEG session immediately after TMS-EEG (modified BRICE questionnaire^37^). In the TMS-EEG-arm, we will take additional blood samples to determine DEX levels around the TMS-EEG sessions (Figure 2).

The discharge criteria will be fulfilled if participants are (i) awake and able to respond appropriately to normal commands, (ii) hemodynamically stable in an upright position for 10 minutes (as deemed by the anesthesiologist), (iii) able to walk unassisted, and (iv) do not have pain, nausea, vomiting, or bleeding from the intravenous and arterial catheter sites.

For successful study completion, participants will receive USD 1000. Remuneration will be incremental and increase towards the end of the study to incentivize participants to complete the study.

#### Outcomes

The primary outcome variable is ‘time to ROR’, defined as the number of minutes from when the DEX infusion is stopped to ROR, ie, five consecutive click responses. The primary outcome is the difference in time to ROR within individuals, ie, between the first and the second study visit. Related exploratory outcomes are differences between females and males, and between study arms (TMS-EEG vs non-TMS-EEG arm).

Secondary outcomes include exploration of DEX-induced state changes of responsiveness and consciousness, as well as characterization of the recovery of higher-order brain functions and autonomic functions following DEX sedation.

To assess responsiveness and consciousness, we will compare presence or absence of behavioral click responses to auditory beeps with EEG signatures suggesting the presence or absence of consciousness (eg, well-modulated alpha, spindles and/or delta waves ≥ 75 μV). In the TMS-EEG arm, we will compute measures of complexity (perturbational complexity index, PCI) to determine the capacity for consciousness. In both study arms, we will also prompt participants to retrospectively report the absence/presence of experiences during sedation, recovery, and TMS-EEG (modified BRICE questionnaire).

To characterize the recovery of higher-order brain functions, we will compare performance scores after ROR and prior to discharge with pre-sedation values (baseline). We will assess verbal fluency (number of words with a given letter per 60 seconds), reaction time and sustained attention (errors of commission and omission), and test digit span (forward and backward). To characterize recovery of autonomic functions, we will determine if and when vital signs (blood pressure, heart rate, SpO2 without oxygen supplementation, galvanic skin response) return to baseline levels, defined as within the 95% confidence interval of same-day baseline values, recorded over a 10-min period.

### Data Collection, Management, and Analysis

#### Data Collection and Management

Starting one week prior to the first study experiment until one week after the second study visit (3 weeks in total, Figure 1), study participants will be asked to complete a paper-based sleep-wake diary and to wear a smartwatch (Fitbit Inspire 2) tracking their sleep-wake rhythm.

Figure 2 depicts an overview of the various assessments and data collection during study visits. We will record EEG, for both the TMS-EEG and non-TMS-EEG arm, using a 64-channel set of TMS-compatible EEG electrodes (EASYCap electrode cap, recorded with BrainAmp DC and BrainVision Recorder, Brain Products GmbH, Gilching, Germany). For TMS-EEG, we will use a neuronavigated TMS system (Nexstim NBT2, Nexstim, Finland), employed with a single-participant MRI (if available). Brain Products sensors and modules will be used for ECG, EMG, plethysmography, and galvanic skin response recording with BrainAmp ExG (Brain Products GmbH, Gilching, Germany). Other vital parameters will be recorded using hospital standard equipment for performing anesthesia.

For prompted, but self-reported sleepiness, we will use a modified version of the Karolinska Sleepiness Scale, for which permission was obtained and granted, ranging from 1 (= fit and fully alert) to 10 (= fell asleep, dozed off).^40^,^41^ We will test verbal fluency using the phonemic test variant with a specified letter (outcome: number of words within 60 s) and the digit span (forward and backward) using randomized numbers. To reduce test–retest learning effects in the verbal fluency test, we will use a different letter [s, c, p] for each verbal fluency test at a study visit and change the sequence between study visits. The sustained attention to response task (SART)^42^ will be used to assess reaction time and sustained attention. During a 4.3-min period, a single number from 1 to 9 will be displayed for 250 ms in a randomized order (25 times per number, 225 times in total) followed by a blank screen (900 ms). Except for number ‘3’, participants will have to respond to each number appearance by button press as quickly as possible.

All electronic research data will be de-identified (key stored in a secure location) and stored on a secured and password-protected network drive or in an institutional Dropbox and/or REDCap database (respecting the Health Insurance Portability and Accountability Act (HIPAA) and with nightly backup). Paper-based data will be stored in a limited-access locked closet.

#### Statistical Methods

To analyze differences in time to ROR, we will use a mixed-effect model (dependent variable: time to ROR; fixed effects: exposure (first, second visit), sex, and study arm; random effect: participant). The null hypothesis is that there are no differences in time to ROR for any of these effects.

The relationship between behavioral responsiveness, EEG signatures and TMS-EEG derived measures of complexity will be explored through initial exploratory data visualization (time series, spectrograms, power spectral density, and spatial distributions) and subsequent statistical analysis. We will calculate the time course of responsiveness probabilities and use moving-window functions to improve temporal comparability between responsiveness, EEG, and TMS-EEG.

To determine the recovery of higher-order brain functions, we will use mixed-effect models using the cognition and performance test outcome variables as the dependent variable (fixed effects: repeated measure (baseline and post-sedation tests), exposure (first, second visit), sex, and study arm; random effect: participant). To determine the recovery of autonomic functions, we will use moving-window functions to determine deviations from the baseline. The 95% confidence interval of baseline values will define deviations from and return to baseline.

### Monitoring

We will monitor and document adverse events during the study visit. The frequency of expected events, managed by a dedicated anesthesiologist in accordance with pre-defined and standardized scenarios (Table 2), and unexpected adverse events will be reported as part of the publication of results. Unexpected or serious adverse events will be reported to the IRB, and if applicable to other authorities, as per protocol and legal regulations. Expedited review will occur for all events meeting the US Food and Drug Administration (FDA) definition of Serious Adverse Events (SAEs) – ie, any events that are fatal, immediately life-threatening, permanently or substantially disabling, or requiring inpatient hospitalization. This also includes any event that a study investigator, or anesthesiologist supporting this study, judges to impose a significant hazard, contraindication, side effect or precaution. The protocol will be stopped for IRB review should one SAE requiring expedited review occur. Reporting to the IRB will be done within 24 hours. Unanticipated problems involving risks to participants or others including adverse events will be reported to the Mass General Brigham Human Research Committee.

## Conclusion

The impact of repeated exposure (potential habituation effect) on the recovery process from DEX sedation is unknown. This pilot trial in 12 healthy individuals aims to investigate the effect of repeated exposure to DEX on time to ROR. REDEX will additionally compare different measures for determining responsiveness and consciousness and characterize the recovery of higher-order brain functions and autonomic functions from DEX exposure.

The findings from REDEX might be limited by the following: First, the study design minimizes the influence of hormonal fluctuations for female sex, but the study does not control for the type of oral hormonal contraception (eg, progesterone only vs estrogen-progesterone), does not measure sex hormone levels, and differences across sex might go beyond hormonal fluctuations. Second, although we do not expect TMS-EEG procedures to promote arousal, due to the paucity of previous evidence it cannot be excluded.

The results of this pilot trial will provide insights into the physiology of DEX sedation, advance our scientific understanding of altered states of consciousness, will inform which future trials might be needed, and provide data for designing such trials accordingly (eg, sample size calculations).

## Data Availability

No new data was generated for this paper.

## Abbreviations

DEX, Dexmedetomidine; ECG, electrocardiogram; EEG, Electroencephalogram; EMG, Electromyography; FDA, US Food and Drug Administration; GCP, Good Clinical Practice; HIPAA, Health Insurance Portability and Accountability Act; ICU, Intensive Care Unit; IRB, Institutional Review Board; LOR, Loss of responsiveness; MGB, Mass General Brigham; MGH, Massachusetts General Hospital; NREM, Non-Rapid Eye Movement Sleep; OR, Operating room(s); PCI, Perturbational Complexity Index; REDEX, REcovery from DEXmedetomidine-induced unresponsiveness; ROR, Return of responsiveness; SART, Sustained attention to response task; SPIRIT, Standard Protocol Items: Recommendations for Interventional Trials; SPL, Sound Pressure Level; TCI, Target-controlled infusion; TMS, Transcranial magnetic stimulation.
